# Early Detection of Sepsis With Machine Learning Techniques: A Brief Clinical Perspective

**DOI:** 10.3389/fmed.2021.617486

**Published:** 2021-02-12

**Authors:** Daniele Roberto Giacobbe, Alessio Signori, Filippo Del Puente, Sara Mora, Luca Carmisciano, Federica Briano, Antonio Vena, Lorenzo Ball, Chiara Robba, Paolo Pelosi, Mauro Giacomini, Matteo Bassetti

**Affiliations:** ^1^Infectious Diseases Unit, San Martino Policlinico Hospital – IRCCS for Oncology and Neurosciences, Genoa, Italy; ^2^Department of Health Sciences (DISSAL), University of Genoa, Genoa, Italy; ^3^Department of Informatics Bioengineering, Robotics, and Systems Engineering (DIBRIS), University of Genoa, Genoa, Italy; ^4^Anaesthesia and Intensive Care, San Martino Policlinico Hospital – IRCCS for Oncology and Neurosciences, Genoa, Italy; ^5^Department of Surgical Sciences and Integrated Diagnostics (DISC), University of Genoa, Genoa, Italy

**Keywords:** sepsis, machine learning, artificial intelligence, early diagnosis, supervised learning, unsupervised learning

## Abstract

Sepsis is a major cause of death worldwide. Over the past years, prediction of clinically relevant events through machine learning models has gained particular attention. In the present perspective, we provide a brief, clinician-oriented vision on the following relevant aspects concerning the use of machine learning predictive models for the early detection of sepsis in the daily practice: (i) the controversy of sepsis definition and its influence on the development of prediction models; (ii) the choice and availability of input features; (iii) the measure of the model performance, the output, and their usefulness in the clinical practice. The increasing involvement of artificial intelligence and machine learning in health care cannot be disregarded, despite important pitfalls that should be always carefully taken into consideration. In the long run, a rigorous multidisciplinary approach to enrich our understanding in the application of machine learning techniques for the early recognition of sepsis may show potential to augment medical decision-making when facing this heterogeneous and complex syndrome.

## Introduction

Sepsis, defined as a life-threatening organ dysfunction caused by a dysregulated host response to infection, is a major cause of death worldwide (1–5).

Sepsis is a complex and evolving concept: (i) complex, because it involves two different actors (the infection and the host response) and their relative contribution to the organ damage may vary across patients and over time in the same patient (6, 7); (ii) evolving, because various definitions have been developed and adopted over the last years, reflecting the complexity of the elusive concept to be defined. Although the evolution of the concept of sepsis could reflect a positive trajectory aimed to provide more precise definitions for both clinical and research purposes, the long-lasting process of defining sepsis has led to the use of different terminology and definitions across research studies (8, 9). As a consequence, this has reduced the comparability of study results, and also hindered a smooth development of predictive models of sepsis. Nonetheless, the development of good predictive models of sepsis remains a timely topic, since it would help clinicians to readily identify patients at higher risk of (or likely to already have) sepsis, thereby allowing close monitoring and/or early treatment (thereby potentially reducing mortality) (10).

Prediction of sepsis through the use of machine learning models has gained particular attention over the past few years (11–17). In the present perspective, we provide a brief clinician-oriented vision on some relevant aspects concerning the use of machine learning predictive models for the prediction of sepsis in the daily practice. In particular, although predictive models may be developed for either prediction *sensu stricto* or early detection (i.e., as early diagnostic tools), we will mainly focus on the ability of these tools to early detect patients with sepsis.

## Methods

On 25 July 2020, we performed a PubMed search using the following key words: “sepsis” AND “machine learning.” Reference lists and abstracts of the pre-screened 189 studies were manually checked, using an iterative approach, and selected for potential eligibility. Selected full texts and pertinent references were further reviewed, with the ultimate decision on inclusion being based upon authors' judgment. Of note, additional searches through PubMed and Google Scholar performed on 23 December 2020 led to the evaluation of 18 additional full texts for potential inclusion. The final text was structured in the following sections: (i) brief introduction on prediction of clinical events through machine learning models and the need for a multidisciplinary approach; (ii) the controversy of sepsis definition and its influence on the development of prediction models; (iii) the choice and availability of input features; (iv) the measure of the model performance, the output, and their usefulness for clinicians; (v) limitations and conclusions.

### Brief Introduction on Prediction of Clinical Events Through Machine Learning Models and the Need for a Multidisciplinary Approach

Through machine learning algorithms, computers are conferred the ability to learn from data (18, 19). Conceptually, machine learning, which is a branch of artificial intelligence, is different from standard computer expert systems for helping clinicians in daily decision making (20). Indeed, the latter are explicitly programmed to perform a given task, whereas machine learning algorithms are more generally programmed to find out associations. For example, in supervised learning, an outcome (e.g., sepsis), is predicted through calculations starting from input features (e.g., patient demographic and clinical characteristics). Notably, the outcome and the input of machine learning algorithms may also represent the dependent and independent variables of classical statistical predictive models. Not surprisingly, there is an important conceptual overlap between classical statistics and machine learning techniques (21). A difference in their continuum may rely on the fact that some machine learning algorithms could be able to find out composite features not easy or impossible to be defined by humans. In turn, this may improve the accuracy of prediction (22). However, this also fuels the issue of the interpretability of the model, with computations that may become less transparent and not completely explainable once results are produced (23). In turn, this may hamper recognition of biases, thereby leading to additional ethical and legal implications connected to the use of machine learning techniques in healthcare (24–26).

In addition, it should be also taken into consideration that the field of machine learning is closely connected with the concept of “big data,” since, at least in general, the possibility of including complex features in prediction models requires far larger samples than those usually employed in classical prediction studies (22, 27). Therefore, the possible future availability of large datasets of medical data from electronic medical records (EMR), laboratory databases, and vital signs monitors will inevitably require a multidisciplinary approach. This would guarantee standardized extraction of data, data security, interpretability or sufficient explanation of employed machine learning models, extrapolation of useful outputs from a clinical perspective, and full compliance to all the most updated ethical and legal laws and regulations. This will also apply to the prediction of sepsis, both for research studies and for real-time prediction at the bedside in the daily clinical practice.

### The Controversy of Sepsis Definition and Its Influence on the Development of Prediction Models

According to the recent Sepsis-3 criteria, sepsis is formally defined as an acute increase of ≥2 points in the Sequential Organ Failure Assessment (SOFA) score, consequent to a suspected or proven infection (1). Increases in the SOFA score, detected through monitoring of laboratory and clinical parameters, reflect possible impairments in cardiovascular, respiratory, renal, hepatic, coagulation, and neurological systems (28). This is in line with the novel definition of sepsis as a life-threatening and progressive organ dysfunction. Previously, sepsis was defined as the presence of a systemic inflammatory response syndrome (SIRS) caused by a proven or suspected infection and based on laboratory and clinical parameters (white blood cell count, respiratory rate, heart rate, and body temperature) that could be altered before manifestation of sepsis-related organ dysfunction (29). However, this previous, more general definition, lacked specificity, since SIRS may be present in several non-infectious conditions. On the other hand, it has very high sensitivity because of the large denominator of patients with SIRS (although some patients with organ dysfunction due to infection may not present with SIRS, and a few cases may still be missed) (30). Overall, the novel definition offers a better performance than the previous one for identifying septic patients at higher risk of mortality in intensive care units (ICU) (1). However, a better prediction of mortality does not inherently reflect the best timing for intervention. For example, it still remains unclear the optimal timing for starting antimicrobial therapy. More specifically, while it is intuitive to initiate antimicrobials if sepsis is first recognized at the time of organ dysfunction (according to the novel sepsis definition), what if the patient is already suspected to have sepsis before developing any organ dysfunction (according to previous sepsis definitions)? Can we wait, or should we initiate antimicrobials immediately? Notably, there is not a high-level evidence-based response to this question yet, and the choice is usually made upon clinical judgment at the patient's bedside, on a case-by-case basis.

However, this is hardly reproducible for defining what should be predicted by both classical statistics and machine learning models to support clinicians in the administration of antimicrobials at the best time to reduce mortality of sepsis, and, at the same time, without administering antimicrobials indiscriminately to all patients with SIRS but no sepsis, in line with antimicrobial stewardship principles. Prediction of sepsis through machine learning techniques is not exempt from this unresolved controversy, as reflected by the various definitions of sepsis employed in the different models available in the literature (see Table 1) (85). Against this backdrop, it is worth noting that the possible solution of using expert physicians' judgement for labeling sepsis cases as gold standard for model training may not resolve the issue because of suboptimal agreement across physicians (86). A reasonable alternative strategy explored by some authors may be the use of unsupervised machine learning techniques (i.e., by recognizing patterns in the data without a labeled outcome as in supervised learning) for the identification of novel phenotypes of sepsis based on clinical and laboratory values (77, 87, 88). This could help in the identification of specific subgroups of patients to be included in dedicated studies (preferably randomized clinical trials) to assess the impact of early antimicrobial therapy (77, 89, 90). Furthermore, this would allow to use the trial outcome as a measure of *a posteriori* accuracy of sepsis classification based on the phenotypes identified with machine learning techniques.

**Table 1:** Characteristics of selected observational studies on the use of machine learning for the prediction/early diagnosis of sepsis^*^.

**References**	**Population**	Sample size^**^	**Input features (or data on which input features are computed)**	Type of employed model/platform^***^	**Endpoint**	Output metrics^****^
Thiel et al. (31)	Mixed-ward patients	27,722	Vital signs, laboratory data	RPART^(6)^	Septic shock defined according to ICD-9 codes for acute infection matched to codes for acute organ dysfunction and the need for vasopressors within 24 h of ICU	Proportion of correctly classified patients, PPV, NPV, MCR
Mani et al. (32)	Neonates in NICU	299	Laboratory, clinical and microbiology data available in EMR	SVM^(3)^, NB^(1)^, TAN^(6, 1)^, AODE^(1)^, KNN^(2)^, CART^(6)^, LR^(10)^, RF^(4)^, LBR^(1)^	Sepsis labeled based on antibiotic use, microbiological data, and laboratory data	AUROC, sensitivity, specificity, PPV, NPV
Henry et al. (33)	ICU patients in the MIMIC-II dataset (34)	16,025	Vital signs, laboratory data	Supervised learning based on Cox proportional hazards model^(18)^ (TREWScore)	Sepsis according to SIRS criteria (35) plus relevant ICD-9 coding or clinical note mentioning sepsis	AUROC, sensitivity, specificity
Calvert et al. (36)	ICU patients in the MIMIC-II dataset (34)	1,394	Vital signs, laboratory data, age	*InSight*^®(19)^	Sepsis according to SIRS criteria (35) plus relevant ICD-9 coding	Accuracy, AUROC, sensitivity, specificity
Desautels et al. (37)	ICU patients in the MIMIC-III dataset (38)	22,853	Vital signs, laboratory data, age	*InSight*^®(19)^	Sepsis according to Sepsis-3 definitions (1)	AUROC, APR
Brown et al. (39)	Patients admitted to the ED	226,481	Vital signs, laboratory data, age	NB^(1)^	Severe sepsis according to ICD-9 coding	AUROC, sensitivity, specificity, PPV, NPV
Horng et al. (40)	Patients admitted to the ED	230,936	Vital signs, demographics, and free text notes	SVM^(3)^	Angus Sepsis ICD-9-CM abstraction criteria (41)	AUROC, sensitivity, specificity, PPV
Kam et al. (42)	ICU patients in the MIMIC-II dataset (34)	6,362	Vital signs, a few laboratory data, age	*InSight*^®(19)^, DNN^(12)^	Sepsis according to SIRS criteria (35) plus relevant ICD-9 coding	Accuracy, AUROC, sensitivity, specificity
Liu et al. (43)	Patients between 16 and 80 years admitted to ICU or medical ICU	185	General information, medical history, progress notes, laboratory data	FANN^(11)^, SVM^(3)^, RF^(4)^	Bacterial sepsis labeled by the treating physician found in the medical records	AUC, accuracy, sensitivity
McCoy et al. (44)	Mixed-ward patients	1,328	Vital signs, laboratory data	*InSight*^®(19)^	Severe sepsis according to 2001 definitions (29) and sepsis according to Sepsis-3 definitions (1)	AUROC, sensitivity, specificity
Shashikumar et al. (45)	ICU patients	242	Vital signs, clinical data	Elastic-net LR^(9, 10)^	Sepsis according to Sepsis-3 definitions (1)	Accuracy, AUROC, specificity
Taneja et al. (46)	Mixed-ward patients	444	Vital signs, laboratory data	LR^(10)^, NB^(1)^, SVM^(3)^, AdaBoost^(5)^, RF^(4)^	Eleven categories from never septic to septic shock	AUROC
Nemati et al. (47)	ICU patients	27,527	Vital signs, laboratory data, demographic and clinical data from EMR	AISE algorithm (based on a modified Weibull-Cox proportional hazards model)^(18)^	Sepsis according to Sepsis-3 definitions (1)	Accuracy, AUROC, sensitivity, specificity
Lamping et al. (48)	Pediatric patients admitted to the PICU	807	Laboratory data, clinical variables	RF^(4)^	Sepsis as defined Goldstein et al. (49)	AUC, sensitivity
Mao et al. (50)	Mixed-ward patients	90,353	Vital signs	*InSight*^®(19)^	Sepsis according to 2001 definitions (29)	AUROC, sensitivity, specificity
Kamaleswaran et al. (51)	Critically ill children	493	Continuous high frequency stream of physiologic data	LR^(10)^, RF^(4)^, DCNN^(13)^	Severe sepsis according to criteria modified from Goldstein et al. (49) and Sepanski et al. (52)	Accuracy, sensitivity, specificity, PPV, NPV, LR+, LR–
Saqib et al. (53)	ICU patients in the MIMIC-III dataset (38)	38,270	Vital signs and laboratory data	LR^(10)^, RF^(4)^, LSTM neural networks^(12)^	Sepsis according to ICD coding system [Angus criteria (54)]	F1 score, Mathew's Correlation Coefficient, AUROC, sensitivity, PPV
Faisal et al. (55)	Patients admitted to the ED	57,243	Vital signs, laboratory data	LR^(10)^	Sepsis according to ICD-10 coding system (56)	AUROC, sensitivity, specificity, PPV
van Wik et al., (57)	ICU patients	1,161	Continuous high frequency stream of physiologic data	RF^(4)^, SVM^(3)^, LR^(10)^, MLP^(10)^, RNN^(12)^	Sepsis defined as SIRS plus presence of blood cultures and administration of antibiotics, along with relevant ICD-10 coding	F1 score, accuracy, AUROC, PPV, sensitivity, specificity
Delahanty et al. (58)	Patients admitted to the ED	2,759,529	Vital signs, laboratory data, demographics, medications, nursing notes, key words	Gradient boosting^(5)^	Sepsis according to Rhee clinical surveillance criteria (59)	Alert rate, AUROC, sensitivity, specificity, precision
Van Wik et al. (60)	ICU patients	586	Continuous high frequency stream of physiologic data	RF^(4)^	Sepsis defined as SIRS plus administration of antibiotics, along with relevant ICD-10 coding	F2 score, accuracy sensitivity, specificity, PPV
Kaji et al. (61)	ICU patients in the MIMIC-III dataset (38)	36,176	Laboratory data, demographics, prescribed medications	LSTM neural networks with attention mechanism^(12)^	Sepsis according to SIRS criteria (35) plus relevant ICD-9 coding	AUROC, sensitivity, PPV
Calvert et al. (62)	Multiple datasets	122,672	Vital signs	Gradient-boosted decision treesclassifier^(5)^	Severe sepsis according to SIRS criteria (35) plus relevant ICD-9 coding	Accuracy, AUROC, sensitivity, specificity, PPV, NPV, DOR
Masino et al. (63)	Neonates in NICU	1,188	Routine EMR data	LR^(10)^, NB^(1)^, SVM^(3)^, KNN^(2)^, Gaussian process^(16)^, RF^(4)^, AdaBoost^(5)^, gradient boosting^(5)^	Sepsis defined as positive blood culture or negative blood culture plus administration of antibiotics	AUROC, sensitivity, specificity, PPV, NPV
Barton et al. (64)	Multiple datasets	112,952	Vital signs, age	Gradient boosting^(5)^	Sepsis according to Sepsis-3 definitions (1)	AUROC, sensitivity, specificity. DOR, LR+, LR–
Giannini et al. (65)	Mixed-ward patients	172,700	Vital signs, laboratory data, demographics	RF^(4)^	Severe sepsis according to ICD-9 coding plus positive blood culture plus increased serum lactate or reduced blood pressure	AUROC, sensitivity, specificity, LR+, LR–
Scherpf et al. (66)	ICU patients in the MIMIC-III dataset (38)	Various (see original publication)	Vital signs, laboratory data, age	RNN^(12)^, *InSight*^®(19)^	Severe sepsis according to SIRS criteria (35) plus relevant ICD-9 coding	AUROC, sensitivity, specificity
Schamoni et al. (67)	Surgical ICU patients	620	Clinical data, laboratory data, demographics	Ordinal regression^(15)^	Sepsis defined according to attending physicians' daily judgements of patients' sepsis status	AUROC, features weights
Fagerström et al. (68)	ICU patients in the MIMIC-III dataset (38)	~59,000	Vital signs, laboratory data, medical procedures, medications, journal notes, diagnoses, patient demographics	LSTM neural networks^(12)^	Septic shock according to SIRS criteria (35) plus relevant ICD-9 coding or clinical note mentioning septic shock	AUROC
Le et al. (69)	Mixed-ward pediatric patients	9,486	Vital signs, laboratory data, GCS	Boosted ensembles of decision trees^(5, 6)^	Severe sepsis defined according to Goldstein et al. (49)	AUROC, sensitivity, specificity, DOR
Yee et al. (70)	ICU patients in the MIMIC-III dataset (38)	9,165	Laboratory data, diagnosis and procedures codes according to ICD-9, demographics	BN^(17)^	Septic shock defined according to Kadri et al. (71)	AUROC, sensitivity, specificity, PPV, NPV
Scott et al. (72)	Pediatric patients admitted to the ED and urgent care sites	2,464	Vital signs, demographics, clinical data	Elastic net regularization^(9)^	Septic shock defined according to Goldstein et al. (49)	AUROC, sensitivity, specificity
Dhungana et al. (73)	ICU patients	200	Laboratory data, clinical data, demographics	Computable phenotypes^(6)^	Sepsis labeled by manual data abstraction from EMR	Sensitivity, specificity
Bloch et al. (74)	ICU patients	600	Vital signs	LR^(10)^, SVM^(3)^, neural networks^(11)^	Sepsis according to 1992 definition (35)	AUROC, APR, sensitivity, specificity, PPV, NPV
Choi et al. (75)	Mixed-ward patients	7,743	Laboratory data	LR^(10)^	Sepsis according to ICD-10 coding system	Accuracy, AUROC, sensitivity, specificity, PPV, NPV
Kim et al. (76)	Patients admitted to the ED	49,560	Vital signs, laboratory data, demographics	SVM^(3)^, gradient boosting^(5)^, RF^(4)^, MARS^(14)^, LASSO^(8)^, ridge regression^(7)^	Septic shock according to Sepsis-3 definitions (1)	AUROC, APR, sensitivity, specificity, PPV, NPV
Ibrahim et al. (77)	ICU patients in the MIMIC-III dataset (38)	13,728	Vital signs, laboratory data	RF^(4)^, gradient boosting^(5)^, SVM^(3)^	Manually validated extracted sepsis mention in EMR, overall and according to phenotypes	AUROC, sensitivity, specificity, LR+, LR–
Yuan et al. (78)	ICU patients	1,588	Minute-by-minute collection of vital signs, laboratory data, examination reports, text data, and images	Gradient boosting^(5)^	Sepsis labeled by the in-charge intensivist every day	F1 score, accuracy, sensitivity, specificity, PPV
Lauritsen et al. (79)	Mixed-ward patients	3,126	Vital signs, laboratory data, clinical data, medications, images	Combination of CNN^(13)^ and LSTM neural networks^(12)^	Sepsis labeled by the treating physician based on the 2001 definitions (29)	AUROC, APR, mean average precision, net benefit
Tran et al. (80)	Adult patients with ≥20% TBSA burns	121	Vital signs, laboratory data, and a few clinical variables and clinical scores	Different ML algorithms optimized by MILO	Sepsis status based on 2007 ABA consensus guidelines (81)	Accuracy, AUROC, sensitivity, specificity
Burdick et al. (82)	Adult patients from inpatient wards and emergency department admission	270,438	Vital signs and a few clinical variables	Optimized distributed gradient boosting^(5)^	Severe sepsis according to ICD-9 coding system	AUROC, specificity, accuracy, DOR, LR+, LR–
Bedoya et al. (83)	Patients admitted to the ED	42,979	Patient demographics, vital signs, comorbidities, medications and laboratory data	RNN^(12)^, CR^(8, 18)^, LR^(10)^,RF^(4)^	Sepsis defined as presence of 2 or more SIRS criteria (35), a blood culture order, and at least one element of end-organ failure	AUROC
Abromavičius et al. (84)	ICU patients	40,336	Vital signs, laboratory data, demographics	DT^(6)^, NB^(1)^, SVM^(3)^, Ensemble learners^(5)^	Sepsis labeled according to Sepsis-3 criteria (1)	AUC, APR, accuracy, F-measure, MCC

*ABA, American Burn Association; AISE; artificial intelligence sepsis expert; AODE, averaged one dependence estimators; APR, area under precision-recall curves; AUROC, area under the receiver operating characteristics curve; BN, Bayesian networks; CART, classification and regression trees; CR, Lasso-penalized Cox regression; DC, decision tree; DCNN, deep convolutional neural networks; DNN, deep neural networks; DOR, diagnostic odds ratio; ED, emergency department; EMR, electronic medical record; FANN, Fast artificial neural network; GCS, Glasgow coma scale; ICD-9-CM, International classification of diseases, ninth Revision, clinical modification; ICU, intensive care unit; IPSCC, International Pediatric Sepsis Consensus Conference; KNN, K-nearest neighbors; LASSO, least absolute shrinkage and selection operator; LBR, lazy Bayesian rules; LR, logistic regression; LR+, positive likelihood ratio; LR–, negative likelihood ratio; LSTM, long short-term memory; MARS, multivariate adaptive regression splines; MCR, misclassification rate; MILO, Machine intelligence learning optimizer; MCC, Matthews correlation coefficient; MLP, multi-layer perceptron; NB, naïve Bayes; NICU, neonatal intensive care unit; NPV, negative predictive value; PICU, Pediatric Intensive Care Unit; PPV, positive predictive value; RF, random forests; RNN, recurrent neural networks; RPART, recursive partitioning and regression tree; SVM, support vector machine, TAN, tree augmented naïve Bayes; TBSA, total body surface area*.

* *Selected studies based on the literature search performed for the purpose of this narrative review. Not meant to be systematic and exhaustive*.

** *Variously divided in training, validation, and test sets*.

*** *Numbers in parentheses refer to specific boxes of Supplementary Figure 1, which provides a brief summary of the characteristics of the different ML model*.

**** *Sensitivity reported at some fixed specificity and vice versa; the same applies to other metrics dependent on cut-offs*.

### The Choice and Availability of Input Features

The increasing use of EMR implies an immediate availability of an electronic form of relevant clinical and laboratory data, that support the development and real-time use of right-aligned predictive models for the early detection of sepsis (91–93). Indeed, these models may continuously update their prediction of sepsis by utilizing the unceasing stream of electronic data from the EMR and/or vital signs monitors (57, 74, 85, 92, 94). A good predictive model may thus allow one to correctly classify in real time a true case of sepsis in the controversial gray area in between the previous and novel sepsis definitions. Amongst others, two important questions need to be addressed: (i) which and what minimum number of input features should be used for developing a good predictive model for the early detection of sepsis? (ii) once a good predictive model is developed, would an early antimicrobial treatment improve the prognosis of these patients?

Intuitively, there can be no answer to the latter question without first developing a good predictive model. Therefore, the appropriate selection of input features remains paramount. In this regard, it is still unclear which between a parsimonious (even of a few vital and/or laboratory parameters) and an expanded (for example, considering also information from unstructured physicians' free text in EMR notes) selection of input features could be preferable, and where precisely is any possible desirable middle in between these two extremes. In the literature, there are encouraging experiences with the use of a few vital and/or laboratory parameters for the prediction of severe sepsis according to previous definitions, with also a positive impact on survival having been registered in a small but randomized single center clinical trial and in some observational studies (44, 95–97). However, the possibility of employing input features relying on patients' clinical data and medical histories remains attractive if only for attempting a concomitant prediction of etiology (e.g., of multidrug-resistant causative agent based on a combination of previous microbiological isolates and risk factors for multidrug-resistant infections, such as previous antibiotic use), that may impact appropriateness of empirical antimicrobial therapy while waiting for blood cultures results (21). Notably, this paves the way to some important issues regarding the automated extraction of unstructured data from EMR. Examples are the need for standardization of extraction, sufficient accuracy of automated extraction, internal/external validation, and continuous re-calibration of automated extraction over time. Furthermore, besides these technical aspects, the presence of missing data [which is inherent in EMR, since they are not an instrument designed for research purposes (50)] and their potential impact on the uncertainty of prediction should be properly handled.

It should also be considered that a different amount of information is expected between ICU patients (more closely monitored clinically and through laboratory tests) and patients in other wards. Therefore, besides inherent differences in their characteristics, also the different amount of available data may influence both the selection of the best set of features and the predictive performance of models developed for ICU patients and models developed for other populations (e.g., to be used in the emergency department), that may be not interchangeable. Finally, the choice of the input features could also be dictated by the local availability of the necessary infrastructures. For example, lack of validation or ability to properly extract clinical data from the local EMR may only allow the use of laboratory values for building predictive models (manual extraction of additional clinical data from EMR may be unfeasible or extremely time-consuming if large samples are concerned).

### The Measure of the Model Performance, the Output, and Their Usefulness for Clinicians

As shown in Table 1, the model performance is frequently measured in term of area under the receiver operating characteristics curve (AUROC), which provide an aggregate measure of performance. Strictly speaking, the AUROC identifies how well predictions are classified considering both the number of true positive and true negative, i.e., how many true cases of sepsis or true cases of not sepsis have been correctly classified. However, this measure may not inherently reflect the clinical usefulness of the algorithm. Indeed, the global value of AUROC is independent of the chosen cut-off, whereas, in the setting of sepsis, the major interest for comparing models may be in the portion of the curve that maximizes sensitivity (in order not to miss true cases and not to delay a potentially life-saving antimicrobial treatment). This would allow, once a low risk of missing true cases is determined, to compare models in terms of specificity (aiming at reducing the number of false positive cases treated with antimicrobials, thereby reducing useless toxicity and resistance selection). In any case, the definitive proof of usefulness of any classification model should be provided by randomized clinical trials comparing sepsis management based on its use vs. standard identification of sepsis with respect to a clinically relevant primary endpoint (e.g., short-term mortality). Finally, it should be kept in mind that the present perspective was aimed to highlight some issues that may hamper the comparability and extrapolation of results of available models, and not to primarily assess their performance, which is not reported in detail. Nonetheless, we think it may be of interest to highlight the wide heterogeneity of model results. For example, the best AUROC for the prediction of sepsis ranged from 0.68 to 0.99 in a recent systematic review, whereas sensitivity and specificity were inconsistently reported to allow proper comparison (92).

Another important aspect is the output of the model. While sepsis is the outcome (or dependent variable), the output of the model may be different according to the type of model (for a brief summary of the technical characteristics of different ML models employed in available studies of sepsis prediction, see Supplementary Figure 1) (98). For example, the output of logistic regression (but also, for example, of a sigmoid function-based output layer a of neural network), intended for a single patient, is his/her probability of experiencing the outcome (in this case having sepsis or not), based on his/her input features and the calculations of the trained predictive algorithm. Let's say, for example, that the algorithm calculates for a novel given patient, based on their clinical and laboratory data, a 40% probability of having sepsis. In terms of usefulness, this probability provided to doctors may be a clinically understandable value that can be easily weighted in the balance when deciding whether or not to administer antimicrobials, possibly also improving acceptance of the implementation of machine learning-based sepsis alerts in daily practice (99). However, clinicians should also be aware of the limitations of the model, in order to further improve an appropriate understanding and use of the output. In this regard, there are no standardized directives on the optimal way to present model pitfalls to clinicians together with the model output, although “model facts” labels have started to be proposed (100).

## Limitations and Conclusions

Given the narrative (and not systematic) nature of this brief narrative perspective, some original works may have not been included, a fact that, together with the lack of an in-depth description of computational aspects of the different algorithms, may represent major limitations of the present paper. Nonetheless, our intention was to provide a brief perspective for clinicians by addressing some topics of clinical interest concerning the application of machine learning techniques for the early detection of sepsis in the daily clinical practice. For this reason, we feel an extended focus on technical aspects would have been beyond the scope of the present manuscript.

In conclusion, the use of predictive tools based on machine learning may support medical decision-making by providing novel elements to improve the correct and early identification of patients with sepsis. Although at the present time it cannot be said yet whether or not this will ultimately improve patient survival and relevant antimicrobial stewardship outcomes, the increasing involvement of artificial intelligence and machine learning in health care cannot be ignored. In the long run, a rigorous multidisciplinary approach to enrich our understanding of the application of machine learning techniques to the early recognition of sepsis may be worth the trip and truly augment medical decision-making when facing this heterogeneous and complex syndrome.

## Data Availability Statement

The original contributions presented in the study are included in the article/Supplementary Material, further inquiries can be directed to the corresponding author.

## Author Contributions

DG made substantial contributions to the study concept and design, first drafting of the manuscript, and critical revision of the manuscript for important intellectual content. AS, MG, PP, SM, LC, CR, LB, and MB made substantial contributions to the study concept and design and critical revision of the manuscript for important intellectual content. FD, AV, and FB made substantial contributions to the literature review and critical revision of the manuscript for important intellectual content. All authors read and approved the final manuscript.

## Conflict of Interest

The authors declare that the research was conducted in the absence of any commercial or financial relationships that could be construed as a potential conflict of interest.
