# Supplementary material for: Early Detection of Sepsis With Machine Learning Techniques: A Brief Clinical Perspective
Source: Front Med (Lausanne). 2021 Feb 12;8:617486. doi: 10.3389/fmed.2021.617486 (PMC7906970; doi:10.3389/fmed.2021.617486)
Supplement: Supplementary file 1 [file Data_Sheet_1.PDF]

Classification

The operation of assigning a label or class to a given unlabeled point.

Ensemble Classification

The combination of the outputs of multiple base classifiers trained on different data subsets creates an ensemble classifier. Depending on how the training sets are selected and on the stability of the base classifiers, ensemble classifiers can help reducing the variance and the bias and this lead to a better overall performance.

Regression

The regression task is to predict the value of a (real-valued) dependent variable given a set of independent ones. The goal is to learn a function that applied to the input points returns the predicted response value. Regression is also a supervised learning approach, so it is necessary a training dataset, comprising input points together with their true response values, to learn the model parameters. After the training phase, the model can be used to predict the response for new test points.

1: Naive Bayes — The (full) Bayes classifier is based on the homonymous theorem with the aim of predicting the class as the one that maximizes the posterior probability. The joint probability density function for each class is modelled via a multivariate normal distribution. The naive Bayes classifier is a simplified version because it assumes that attributes are independent, but it is still powerful for many applications. Both are probabilistic classification approaches.

2: Knn — K nearest neighbours (KNN) is a probabilistic classification method that uses a non-parametric approach to estimate the density. Because K is fixed, the KNN classifier predicts the class of a given input as the majority voting class among its K nearest neighbours distribution.

3: Svm — The support vector machine (SVM) is a supervised method based on maximum margin linear discriminants. The main aim is to find the optimal hyperplane that maximizes the gap or margin between the classes. To compute the maximum margin search, the loss function in the SVM method is the Hinge Loss.

4: Rf — Random forest (RF) is an ensemble of K decision tree classifiers created from a different bootstrap sample. The trees are built by sampling a random subset of the attributes at each internal node in the decision tree. The random sampling of the attributes reduces the correlation between the trees in the ensemble.

5: Boosting — Boosting classifiers are a group of machine learning algorithms that combine many weak learning models (classifiers that are only slightly correlated to the true classifier) together to create a strong predictive model. It relies on the intuition that the best possible next model, when combined with previous models, minimizes the overall prediction error.

6: Decision Tree — A decision tree is a combination of internal nodes representing the decisions corresponding to the hyperplanes or split points and leaf nodes representing regions or partitions of the data space, which are labeled with the majority class. The subset of data points that lie in a region characterize that specific region.

7: Ridge — Ridge is a regression method with regularization (in order not to fit the data exactly, aiming at reducing overfitting). Instead of trying to simply minimize the squared residual error, a regularization term involving the squared norm of the weight vector (L_2) is added.

8: Lasso — Lasso is the acronym for least absolute selection and shrinkage operator. It is a regression method with regularization that instead of using the L_2 or Euclidean norm for weight regularization as in ridge regression, it uses the L_1 norm for regularization (also known as Manhattan Distance, it is the absolute value of the weight vector). The main advantage of using L_1 regression is that it can drive the coefficients to zero and so when there are many predictor attributes this results in a more interpretable model. This method is used to compute dimensionality reduction.

9: Elastic Net — It is a regularization method that combines the L_1 and L_2 penalties of the Lasso and Ridge methods in order to make noise less influent.

10: Logistic Regression — Logistic regression is a classification technique that, given a set of predictor attributes or independent variables and given a categorical response, predicts the probability of the response variable values based on the independent variables.

Neural Network — Artificial neural networks are composed of abstract neurons that try to imitate real neurons at a very high level. They can be described via a weighted directed graph, with each node representing a neuron, and each directed edge representing a synaptic to dendritic connection between two neurons. The weight of the edge denotes the synaptic strength. The type of activation function used to generate an output and the architecture of the network in terms of how the nodes are interconnected characterize the neural network.

- **11:Mlp** — Multilayer Perceptrons (MLP) are feed-forward networks in which the information flows in only one direction, specifically from the input layer to the output layer via the hidden layers.
- **12:Rnn** — Recurrent Neural Networks (RNN) are a specific type of neural networks, called deep neural networks, that include feedback from one layer to another. In other words, RNNs are dynamically driven with a feedback loop between two (or more) layers. This type of networks are ideal for learning from sequence data.
- **13:Cnn** — A Convolutional Neural Network (CNN) is a deep MLP designed to exploit spatial and/or temporal structure in the input data. CNN connects a contiguous or adjacent subset of neurons in one layer, in example L, to a single neuron in the next layer, so L+1. Different sliding windows comprising contiguous subsets of neurons in layer L connect to different neurons in layer L+1.

14: MARS — The Multivariate Adaptive Regression Splines (MARS) is a form of non-parametric regression technique that automatically models nonlinearities and interactions between variables.

15: Ordinal Regression — Ordinal Regression is a type of regression analysis used for predicting an ordinal variable, for example those variables whose value exists on an arbitrary scale where is significant only the relative ordering between different values.

16: Gaussian Process — A Gaussian process is a generic supervised learning method designed to solve regression and probabilistic classification problems. A machine-learning algorithm that involves a Gaussian process uses lazy learning and a kernel function to predict the value for a new unknown point from training data.

17: Bayesian Network — It is a probabilistic graphical model that represents a set of variables and their conditional dependencies. It can be used to take an occurred event and predict the likelihood that any one of several possible known causes contributed as a factor.

18: Proportional hazards model — Proportional hazards models belong to the class of survival models. Their main aim is to correlate the time that passes, before a specific event occurs, to one or more covariates that may be associated with that quantity of time. A unit increasing in a covariate generates a multiplicative effect with respect to the hazard rate.

19: Insight® — InSight® is based on gradient boosted trees to compute a risk in real-time. Gradient tree boosting is an ensemble technique which combines the results from multiple weak decision trees in an iterative fashion.
